# Rotationally inelastic dynamics of LiH (X^1^Σ^+^, *v* = 0) in collisions with Ar: State-to-state inelastic rotational rate coefficients

**DOI:** 10.1186/2193-1801-3-188

**Published:** 2014-04-14

**Authors:** Aliou Niane, Cheikh Amadou Bamba Dath, Ndèye Arame Boye Faye, Kamel Hammami, Nejm-Eddine Jaidane

**Affiliations:** Laboratory of Atoms Lasers, Department of Physics, Faculty of Science and Techniques, University Cheikh Anta Diop of Dakar, Dakar, Senegal; Laboratory for Atomic Molecular Spectroscopy and Applications, Department of Physics, Faculty of Science, University Tunis El Manar, Campus Universities, 1060 Tunis, Tunisia

**Keywords:** PES, CC approach, Collision, Cross-sections, Rate coefficients

## Abstract

A theoretical study of rotational collision of LiH(X^1^Σ^+^,*v* = 0, *J*) with Ar has been carried out. The *ab initio* potential energy surface (PES) describing the interaction between the Ar atom and the rotating LiH molecule has been calculated very accurately and already discussed in our previous work [Computational and Theoretical Chemistry 993 (2012) 20–25]. This PES is employed to evaluate the de-excitation cross sections. The *ab initio* PES for the LiH(X^1^Σ^+^)-Ar(^1^S) Van der waals system is calculated at the coupled-cluster [CCSD(T)] approximation for a LiH length fixed to an experimental value of 3.0139 bohrs. The basis set superposition error (BSSE) is corrected and the bond functions are placed at mid-distance between the center of mass of LiH and the Ar atom. The cross sections are then derived in the close coupling (CC) approach and rate coefficients are inferred by averaging these cross sections over a Maxwell-Boltzmann distribution of kinetic energies. The 11 first rotational levels of rate coefficients are evaluated for temperatures ranging from 10 to 300 K. We notice that the de-excitation rate coefficients appear large in the order 10^−10^ cm^−3^ s^−1^ and show very low temperature dependence. The rate coefficients magnify significantly the propensity toward ∆* J* = −1 transitions. These results confirm the same propensity already noted for the cross sections.

## Introduction

The analysis of atom diatom scattering of molecular collisions shows a field of current interest (Santiago *et* al. 2008; Aguillon *et* al. 2000). The rotational collisions between diatomic molecules and atomic partners give rise to complex energy transfer processes which provide one of the most rigorous tests of high-level *ab initio* potential energy surfaces (Paterson *et* al. 2011; Dagdigian *et* al. 1995, 1997; Eyles *et* al. 2011). The LiH molecule has much interest in atmospherical models (Dalgarno *et* al. 1996; Gianturco *et* al. 1999) thanks to its importance in chemistry of the lithium. These calculations of rate coefficients are stimulated by the studies of Ren *et* al. (2006) which have shown that the high-purity Ar atmosphere at room temperature contribute significantly to the stability of LiH in environment.

The collision dynamics of LiH(X^1^Σ^+^) with rare gas (Rg) has received particular attentions. Theoretically, the electronic structure methods, semi-empirical as well as *ab initio*, have been employed to calculate intermolecular potential energy surfaces to study the LiH in excited rotational levels by collision with the atoms H (Berriche and Tlili 2004; Berriche 2004), He (Gianturco *et* al. 1997a, 1997b; Bodo *et* al. 1998; Forni 1999) and Ne (Lu *et* al. 2000; Feng *et* al. 2004; Feng *et* al. 2005). These studies have shown the weakly van der Waals forces interacting molecular systems of LiH in its ground electronic state with Rg atoms. The competition between the charge transfer processes and the chemical binding have done that the interactions of van der Waals systems represent a critical test for the potential energy surfaces (PESs).

In our previous work for the LiH-Ar system, we have reported the first quantum mechanical close coupling calculations of integral cross sections for transitions between the lower rotational levels of LiH induced by collision with Ar based on the *ab initio* potential energy surface. We have used in all the calculations the *ab initio* coupled-cluster [CCSD(T)] level of theory and with aug-cc-pVQZ Gaussian basis set for the H and Ar atoms and cc-pVQZ Gaussian basis set for the Li atom.

## Interaction potential energy surface

We have computed the interaction PES for the LiH(X^1^Σ^+^)–Ar(^1^S) Van der Waals system using the rigid-rotor approximation and the Jacobi coordinate system in which *r*_e_ is the LiH internuclear distance, *R* the distance from the center of mass (c.m) of LiH to Ar atom, and θ the angle between the two distance vectors. The collinear LiH…Ar geometry corresponds to θ = 0° while the LiH bond length is frozen at the experimental equilibrium geometry of the ground state *r*_e_ = 3.0139 bohr (Huber & Herzberg 1979). Treating all geometries in the *CS* symmetry group, the PES has been computed with the CCSD(T) method (Knowles *et* al. 1993; 2000) as implemented in the MOLPRO2002 package (Werner *et* al. 2009). The H and Ar atoms have been described by the standard aug-cc-pVQZ basis set (Hutson & Green 1994; Smith *et* al. 1979; Lique *et* al. 2007). The Li atom has been described with cc-pVQZ basis set which we have added (1s1p1d1f1g) functions (Dunning 1989; Kendall et al. 1992; Woon and Dunning 1994, 1995). To this basis, we have added a set of (3s3p2d2f1g) bond functions defined by Cybulski and Toczylowski (1999) and placed at mid-distance between the center of mass of LiH and Ar atom. The basis set superposition error (BSSE) has been corrected at all geometries with the counterpoise procedure of Boys and Bernadi (1970). The PES obtained has a global minimum of 525.13 cm^−1^ located at *R* = 5.30 bohr and θ = 180°. The anisotropy of the PES is very large because of the character stems from the electronic structure of the LiH.

The basic input required by the MOLSCAT (Hutson & Green 1994) package used in dynamics calculations, were obtained by expanding the interaction potential in terms of Legendre polynomials as:

From *ab initio* grid containing 19 values of θ, we have been able to include terms up to λ_*max*_ = 18 The standard deviation between the analytical form and the calculated surface remains below 1.0%.

## Results and Discussion

### Rotational cross sections

Using the propagator of Manolopoulos (1986) as implemented in the MOLSCAT quantum mechanical code (Hutson and Green 1994), the scattering cross sections have been calculated with the close coupling approach developed by Arthurs and Dalgarno (1960) for a total energy ranging from 15 to 2500 cm^−1^. The energy steps are 0.1 cm^−1^ below 100 cm^−1^, 0.5 cm^−1^ from 100 to 500 cm^−1^, 1 cm^−1^ from 500 cm^−1^ to 1000 cm^−1^, 10 cm^−1^ from 1000 cm^−1^ to 1500 cm^−1^ and 500 cm^−1^ from 1500 cm^−1^ to 2500 cm^−1^. For the rotational basis sets, we have used *J*_*max*_ = 10 for *E* ≤ 100 cm^−1^, *J*_*max*_ = 15 for 100 < *E* ≤ 1000 cm^−1^ and *J*_*max*_ = 30 for *E* ≥ 1000 cm^−1^. The scattering calculations have been performed to rotational basis sets of adequate size for a good accuracy of the results. The other parameters required as input in MOLSCAT and displayed in Table 1 have been fixed after the convergence tests. However, the maximum values of the total angular momentum JTOT was chosen according to a convergence criterion of the cross sections to within 0.01 Å for diagonal terms and 0.001 Å for off-diagonal ones. For example, we have JTOT = 97, 179, 229 and 317 for the collision energies of 100 cm^−1^, 500 cm^−1^, 1000 cm^−1^ and 2500 cm^−1^ respectively.

**Table 1:** **MOLSCAT parameters used in the present calculations**

INTFLG = 6	STEPS = 20, 10	OTOL = 0.001	DTOL = 0.01
Be = 7.513100 cm^−1^	De = 0.00086170 cm^−1^	Jmax = 10, 15, 30	Rmin = 3.0 bohr Rmax = 30 bohr

**Figure 1:**
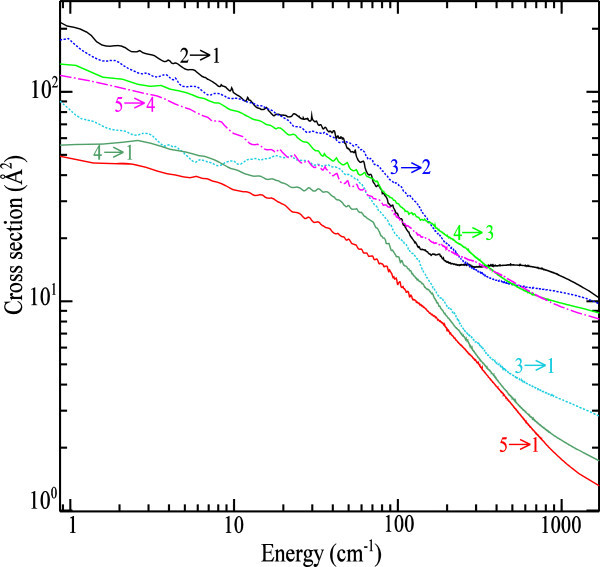
**Rotational de-excitation cross sections of LiH in collision with Ar as a function of the relative kinetic energy.**

The Figure 1 presents the energy dependence of the LiH-Ar collisional de-excitation cross sections for the ∆*J* = *J' – J = −* 1, *J* = 3 → 1, *J* = 4 → 1 and *J* = 5 → 1 rotational transitions. As one can see for collision energy below 200 cm^−1^, this figure illustrates some resonances. These resonances are the *shape* resonances due to the quasi-bound states arising from the trap of Ar atom into the well depth (Smith *et* al. 1979; Christoffel and Bowman 1983) and the *Feshbach* resonances in the vicinity of the opening of a new j level derived to tunneling through the centrifugal energy barrier. These facts have been discussed by Vincent *et* al. (2007).

The de-excitation cross sections decrease with increasing ∆*J* as a function of the kinetic energy. An analysis of this figure shows clearly that the magnitude of the transition 2 → 1 is larger than the others included the transition 1 → 0 in our previous work (Niane *et* al. 2012). The plots of de-excitation cross sections decrease with increasing ∆*J* and have almost the similar behavior. The Figure 1 illustrates the propensity in favor of the transitions ∆*J* = *J' – J = −* 1. This is consistent with similar results by Hammami *et* al. (2009). The features in our recent excitation cross sections (Niane *et* al. 2012) for the transitions 1 → *J* allow understanding the detailed balance equation that relate excitation and de-excitation cross sections.

### Downward rate coefficients

**Figure 2:**
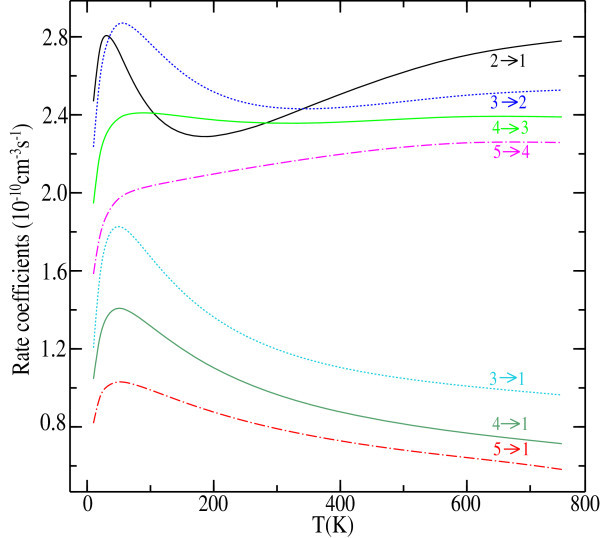
**Calculated downward rate coefficients for the collisions of LiH with Ar for** ***J*** **→ 0 (** ***J*** **= 2 – 5) panel (a) and ∆** ***J*** **= −1 (** ***J’*** **= 1 – 4;** ***J*** **= 2 – 5) and** ***J*** **→ 1(** ***J*** **= 2 – 5) panel (b) transitions as a function of the kinetic temperature.**

The downward rate coefficients are calculated by averaging from rotational cross sections σ_*J→J’*_(*E*_*k*_) over a Maxwell-Boltzmann distribution of kinetic energies *E*_*k*_ following the procedure used in previous works (Hammami *et* al. 2008a, 2008b, 2009; Nkem *et* al. 2009).

where *T* is the kinetic temperature, *μ* = 6.68193048 a.u. is the reduced mass of the LiH-Ar collision partners,  (*k*_B_ is the Boltzmann constant) and *E*_*k*_ = *E – E*_*j*_ is the relative kinetic energy. The Table 2 displays the results at selected temperatures.To better analyse the rates variation with temperature are shown in Figure 2.

**Table 2:** **Downward rate coefficients (given as A(B) = A.10** ^**B**^ **) of rotational levels of LiH in collisions with Ar as a function of kinetic temperature (in units of cm** ^**3**^ **s** ^**−1**^ **)**

Initial	Final	Rate coefficients						
level ***J***	level ***J’***	10 K	30 K	50 K	100 K	150 K	200 K	300 K
1	0	1.4523(−10)	1.4645(−10)	1.4592(−10)	1.5986(−10)	1.7872(−10)	1.9545(−10)	2.2034(−10)
2	0	5.8075(−11)	7.1884(−11)	7.2722(−11)	6.7117(−11)	6.3849(−11)	6.2450(−11)	6.1586(−11)
2	1	2.4694(−10)	2.8061(−10)	2.7242(−10)	2.4286(−10)	2.3069(−10)	2.2910(−10)	2.3796(−10)
3	0	3.9204(−11)	5.6170(−11)	5.7489(−11)	5.0864(−11)	4.4844(−11)	4.0685(−11)	3.5799(−11)
3	1	1.2052(−10)	1.7421(−10)	1.8270(−10)	1.6721(−10)	1.4951(−10)	1.3632(−10)	1.1974(−10)
3	2	2.2353(−10)	2.7512(−10)	2.8660(−10)	2.7651(−10)	2.6171(−10)	2.5181(−10)	2.4369(−10)
4	0	3.5679(−11)	4.6672(−11)	4.7499(−11)	4.2990(−11)	3.8446(−11)	3.4945(−11)	3.0214(−11)
4	1	1.0461(−10)	1.3556(−10)	1.4080(−10)	1.3174(−10)	1.1999(−10)	1.1031(−10)	9.6563(−11)
4	2	1.5181(−10)	1.8957(−10)	1.9925(−10)	1.9695(−10)	1.8754(−10)	1.7818(−10)	1.6299(−10)
4	3	1.9458(−10)	2.2991(−10)	2.3826(−10)	2.4094(−10)	2.3928(−10)	2.3746(−10)	2.3571(−10)
5	0	3.2612(−11)	3.8686(−11)	3.8332(−11)	3.4966(−11)	3.1964(−11)	3.9586(−11)	2.6135(−11)
5	1	8.1900(−11)	1.0025(−10)	1.0301(−10)	9.8940(−11)	9.2976(−11)	8.7593(−11)	7.9068(−11)
5	2	1.2049(−10)	1.3733(−10)	1.4167(−10)	1.4138(−10)	1.3770(−10)	1.3348(−10)	1.2550(−10)
5	3	1.4602(−10)	1.6247(−10)	1.6695(−10)	1.6875(−10)	1.6826(−10)	1. 6712(−10)	1.6388(−10)
5	4	1.5841(−10)	1.8774(−10)	1.9702(−10)	2.0350(−10)	2.0674(−10)	2.0968(−10)	2.1505(−10)
6	0	2.3956(−11)	2.7303(−11)	2.7071(−11)	2.5504(−11)	2.4158(−11)	2.3035(−11)	2.1228(−11)
6	1	5.6627(−11)	6.6954(−11)	6.8777(−11)	6.8459(−11)	6.6913(−11)	6.5130(−11)	6.1593(−11)
6	2	7.8772(−11)	9.0165(−11)	9.3549(−11)	9.6310(−11)	9.6931(−11)	9.6642(−11)	9.4753(−11)
6	3	9.9003(−11)	1.1003(−10)	1.1335(−10)	1.1672(−10)	1.1871(−10)	1.2005(−10)	1.2111(−10)
6	4	1.1464(−10)	1.3069(−10)	1.3559(−10)	1.4003(−10)	1.4277(−10)	1.4518(−10)	1.4885(−10)
6	5	1.4103(−10)	1.6839(−10)	1.7655(−10)	1.8257(−10)	1.8621(−10)	1.9002(−10)	1.9764(−10)
7	0	1.5219(−11)	1.6972(−11)	1.7037(−11)	1.6840(−11)	1.6642(−11)	1.6479(−11)	1.6070(−11)
7	1	3.6069(−11)	4.1334(−11)	4.2680(−11)	4.4206(−11)	4.5119(−11)	4.5593(−11)	4.5618(−11)
7	2	4.8848(−11)	5.5593(−11)	5.8314(−11)	6.2473(−11)	6.5332(−11)	6.7306(−11)	6.9356(−11)
7	3	6.2249(−11)	6.9436(−11)	7.2594(−11)	7.7704(−11)	8.1503(−11)	8.4507(−11)	8.8581(−11)
7	4	7.5679(−11)	8.5248(−11)	8.9100(−11)	9.4838(−11)	9.9007(−11)	1.0250(−10)	1.0795(−10)
7	5	9.5470(−11)	1.0918(−10)	1.1370(−10)	1.1934(−10)	1.2351(−10)	1.2726(−10)	1.3370(−10)
7	6	1.3671(−10)	1.6099(−10)	1.6733(−10)	1.7162(−10)	1.7462(−10)	1/7817(−10)	1.8590(−10)
8	0	8.8089(−12)	9.7013(−12)	9.9159(−12)	1.0294(−11)	1.0690(−11)	1.1032(−11)	1.1471(−11)
8	1	2.1408(−11)	2.4022(−11)	2.5081(−11)	2.7118(−11)	2.8920(−11)	3.0395(−11)	3.2349(−11)
8	2	2.9185(−11)	3.2861(−11)	3.4919(−11)	3.9058(−11)	4.2518(−11)	4.5355(−11)	4.9356(−11)
8	3	3.7481(−11)	4.1756(−11)	4.4470(−11)	4.9984(−11)	5.4442(−11)	5.8151(−11)	6.3736(−11)
8	4	4.6666(−11)	5.2136(−11)	5.5420(−11)	6.1976(−11)	6.7113(−11)	7.1358(−11)	7.8001(−11)
8	5	5.9281(−11)	6.6721(−11)	7.0434(−11)	7.7459(−11)	8.3015(−11)	8.7657(−11)	9.5109(−11)
8	6	8.2936(−11)	9.3863(−11)	9.7797(−11)	1.0386(−10)	1.0884(−10)	1.1334(−10)	1.2110(−10)
8	7	1.3408(−10)	1.5491(−10)	1.6003(−10)	1.6371(−10)	1.6652(−10)	1.6996(−10)	1.7762(−10)
9	0	4.7020(−12)	5.2283(−12)	5.4786(−12)	6.0201(−12)	6.5558(−12)	7.0524(−12)	7.8370(−12)
9	1	1.1801(−11)	1.3287(−11)	1.4146(−11)	1.6069(−11)	1.7895(−11)	1.9553(−11)	2.2164(−11)
9	2	1.6467(−11)	1.8651(−11)	2.0197(−11)	2.3731(−11)	2.6917(−11)	2.9747(−11)	3.4258(−11)
9	3	2.1330(−11)	2.4051(−11)	2.6216(−11)	3.1200(−11)	3.5466(−11)	3.9152(−11)	4.5064(−11)
9	4	2.6912(−11)	3.0344(−11)	3.3040(−11)	3.9293(−11)	4.4497(−11)	4.8862(−11)	5.5809(−11)
9	5	3.4467(−11)	3.8889(−11)	4.1962(−11)	4.9054(−11)	5.5023(−11)	6.0004(−11)	6.7897(−11)
9	6	4.6934(−11)	5.2930(−11)	5.6296(−11)	6.3612(−11)	6.9894(−11)	7.5266(−11)	8.3952(−11)
9	7	7.1389(−11)	8.0616(−11)	8.4337(−11)	9.1006(−11)	9.6683(−11)	1.0180(−10)	1.1054(−10)
9	8	1.2869(−10)	1.4711(−10)	1.5162(−10)	1.5584(−10)	1.5917(−10)	1.6291(−10)	1.7084(−10)
10	0	2.4197(−12)	2.7180(−12)	2.9313(−12)	3.4324(−12)	3.9173(−12)	4.3837(−12)	5.1889(−12)
10	1	6.2689(−12)	7.1015(−12)	7.7515(−12)	9.3244(−12)	1.0841(−11)	1.2286(−11)	1.4776(−11)
10	2	8.9852(−12)	1.0238(−11)	1.1346(−11)	1.4105(−11)	1.6691(−11)	1.9095(−11)	2.3229(−11)
10	3	1.1774(−11)	1.3402(−11)	1.4980(−11)	1.8997(−11)	2.2613(−11)	2.5840(−11)	3.1273(−11)
10	4	1.4982(−11)	1.7035(−11)	1.9048(−11)	2.4279(−11)	2.8911(−11)	3.2908(−11)	3.9456(−11)
10	5	1.9199(−11)	2.1785(−11)	2.4145(−11)	3.0330(−11)	3 ?5872(−11)	4.0614(−11)	4.8262(−11)
10	6	2.5855(−11)	2.9221(−11)	3.1888(−11)	3.8707(−11)	4.4906(−11)	5.0278(−11)	5.8978(−11)
10	7	3.7560(−11)	4.2255(−11)	4.5290(−11)	5.2547(−11)	5.0100(−11)	6.4872(−11)	7.4413(−11)
10	8	6.1122(−11)	6.8648(−11)	7.2240(−11)	7.9634(−11)	8.6010(−11)	9.1702(−11)	1.0130(−10)
10	9	1.2147(−10)	1.3727(−10)	1.4152(−10)	1.4706(−10)	1.5149(−10)	1.5590(−10)	1.6445(−10)

We notice that the downward rate coefficients depend on low temperature. This effect of temperature dependence has been seen by Taylor and Hinde (2005) at little temperature, they explain that the lack of dependence is indicated of downward mechanism in which attractive collisions dominate the energy transfer process for ion-molecule processes. The LiH-Ar system is very attracted when the Ar atom is near the lithium end of LiH. The Figure 2 shows the propensity toward ∆*J* = − 1 transitions. This result confirms the same propensity observed with the cross sections and remains an important consequence of atmospherical chemistry.

**Figure 3:**
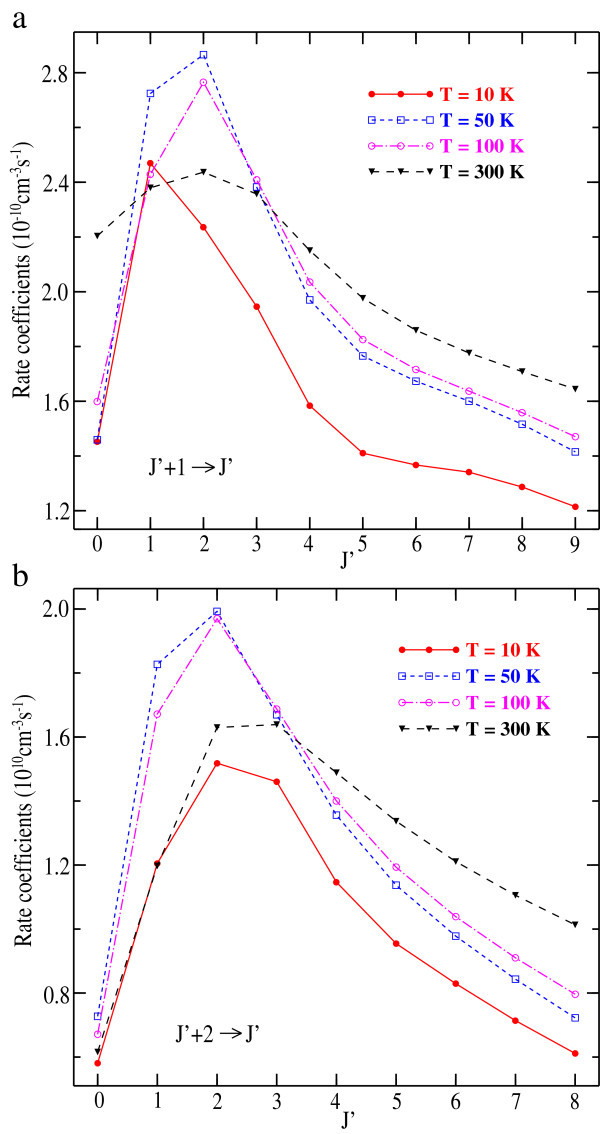
**Calculated downward rate coefficients for the collisions of LiH with Ar for** ***J’*** **+ 1 →** ***J’*** **and** ***J’*** **+ 2 →** ***J’*** **transitions for selected kinetic temperature.**

**Figure 4:**
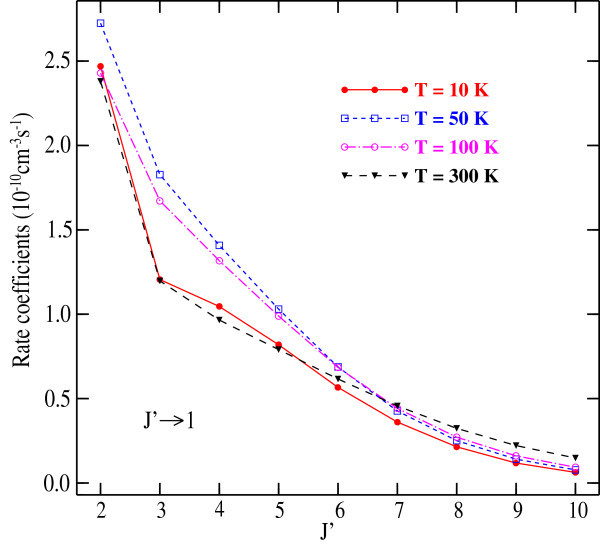
**Calculated downward rate coefficients for the collisions of LiH with Ar for panel (a)** ***J’*** **→ 0 and panel (b)** ***J’*** **→ 1transitions for selected kinetic temperature.**

We report in Figures 3 and 4 the downward rate coefficients as a function of *J* for selected ∆*J* = −1, −2 and *J* → 1 transitions respectively. Except the ∆*J* = −1 transition at 10 K, the plots of rate coefficients exhibit the same trends and decrease with increasing *J*’ from *J*' = 2. For *J* → 1, the downward rate coefficients decrease with increasing *J* and the gap between the plots narrow considerably. In addition, the collision rate coefficients reflect the similar behavior with the general trends observed earlier for HCP-He (Hammami *et* al. 2008b) and HCP-H_2_ (Hammami *et* al. 2008c) systems.

## Conclusion

In this work, using the *ab initio* PES LiH(X^1^Σ^+^)–Ar(^1^S) van der Waals system computed in our previous work (Niane *et* al. 2012), we have obtained results of a quantum mechanical close coupling calculation of integral cross sections for lower rotational levels. By averaging the cross sections over a Maxwell-Boltzmann distribution of kinetic energies, we have inferred the downward rate coefficient for the lowest 11 levels.

The downward rate coefficients at 300 K for the transitions 1 → 0, 2 → 1 and 3 → 2 are estimated respectively at 2.5664 10^−10^, 2.7792 10^−10^ and 2.5272 10^−10^ cm^3^s^−1^. It is obvious that these results may be useful for the atmospherical chemistry as well as for experiments. Finally, encouraged by this result, we will be undertaking the study of the spectroscopy of complex and the vibrational dependence of potential energy surface which is crucial for the diatomic molecular.
